# Possible Links Between PM_2.5_-Induced NLRP3 Inflammasome Activation and Its Role in Lung Cancer Progression and Drug Resistance

**DOI:** 10.3390/ijms27198931

**Published:** 2026-10-08

**Authors:** Thanaphat Worawichitchaikun, Pitchapa Wantanachaisaeng, Teerapat Teeppaibul, Prasit Suwannalert

**Affiliations:** 1 Department of Pathobiology, Faculty of Science, Mahidol University, Bangkok 10400, Thailand; thanaphat.worawich@gmail.com (T.W.); pitchapa.wantana@gmail.com (P.W.); teerapat.teep@gmail.com (T.T.); 2 Pathobiology Information and Learning Center, Department of Pathobiology, Faculty of Science, Mahidol University, Bangkok 10400, Thailand

**Keywords:** PM_2.5_, NLRP3 inflammasome, oxidative stress, lung cancer, drug resistance

## Abstract

The rising incidence of lung cancer is increasingly linked to fine particulate matter (PM_2.5_) exposure, highlighting its role as a risk factor of lung carcinogenesis. However, the molecular mechanisms behind PM_2.5_ and the disease remain incompletely understood. Exposure to PM_2.5_ triggers inflammatory processes, primarily via ROS overproduction, which activates the NLRP3 inflammasome, a cytosolic NOD-like pattern recognition receptor protein complex that cleaves caspase-1 and drives maturation of pro-interleukin-1β (IL-1β) and pro-interleukin-18 (IL-18), leading to chronic pulmonary inflammation. Based on current evidence, this review displays PM_2.5_-induced ROS upregulation as an essential trigger of NLRP3 inflammasome activation in lung cancer development and progression and hypothesises that the signalling of NLRP3 may contribute to molecular pathways associated with lung cancer drug resistance. There have been uses of NLRP3 inhibitors in clinical trials of different inflammatory diseases, but none has yet reached clinical testing in oncology. We propose the potential of NLRP3 as a possible prognostic marker and a therapeutic target for PM_2.5_-associated lung cancer drug resistance.

## 1. Current Overview of Lung Cancer Incidence

Cancer is a disease in which human cells develop uncontrollable cell growth and have the potential to progressively spread to other parts of the body. One of the most common carcinomas, and in fact the leading cause of cancer-related deaths in recent years, is lung cancer [1,2]. According to information from the Global Cancer Observatory (GLOBOCAN) in 2022, lung cancer is the leading cause of global cancer incidence and mortality, representing 12.4% of all new cancer diagnoses and 18.7% of all cancer-related deaths [3]. There were more than 2 million new cases of lung cancer and approximately 1.8 million lung cancer-related deaths in 2022, with the Asian region bearing a disproportionate share, accounting for nearly 60% of the global mortality burden [3,4]. Moreover, recent demographic shifts reveal that lung cancer incidence is increasingly characterised by a younger age at diagnosis alongside a narrowing sex gap [5]. While lung cancer was historically dominated by older males, declining male smoking rates coupled with persistent environmental exposure and non-tobacco risk factors in females have driven down male incidence while female rates have surged [4,5].

**Figure 1:**
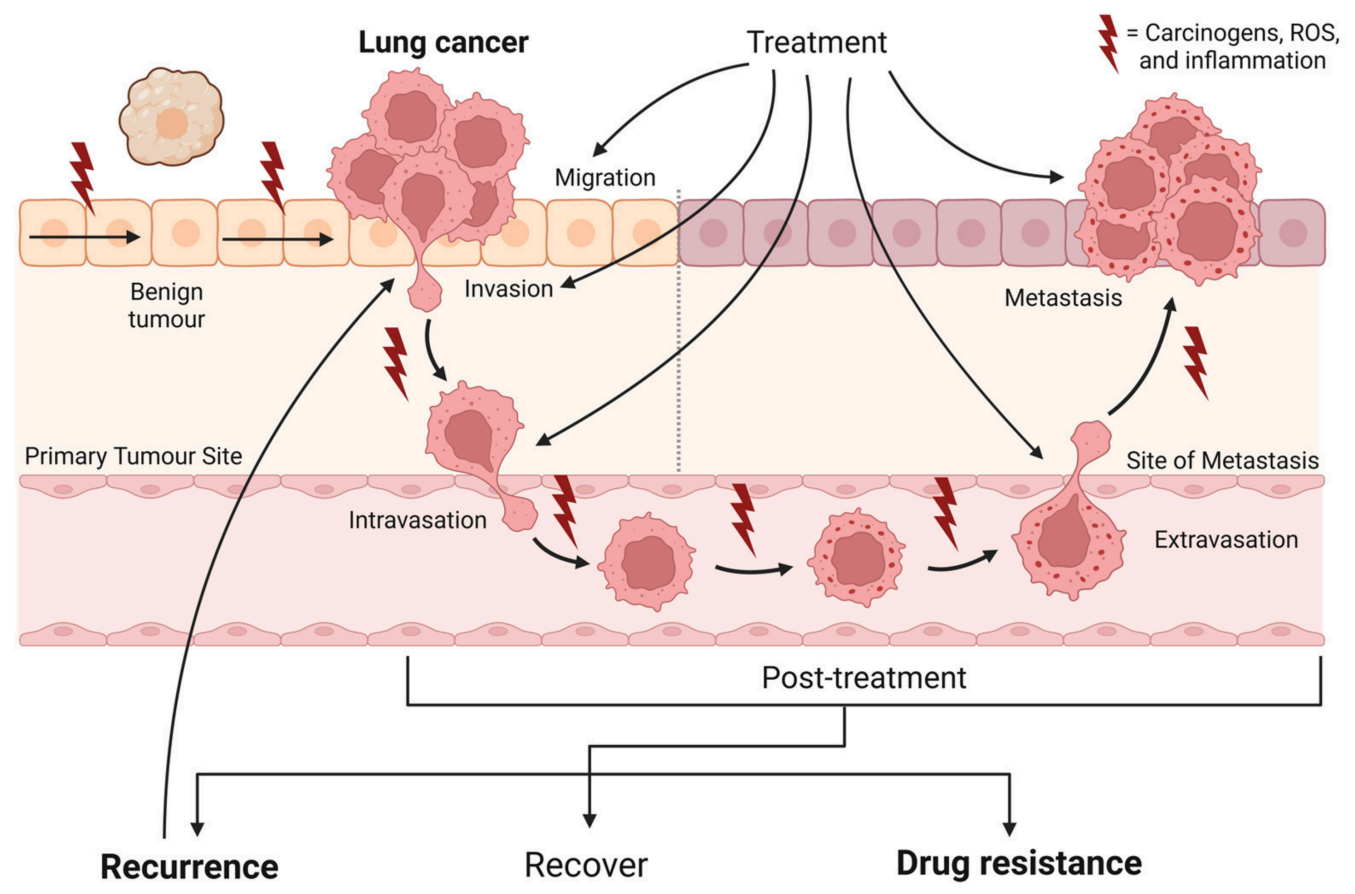
The schematics representing the progression of lung cancer influenced by contributing factors including carcinogens, reactive oxygen species (ROS), and related inflammation. The schematics of treatment application, which influence cancer survival, are also shown as causing patients to either enter remission or develop drug resistance or recurrence. (Created in BioRender. Worawichitchaikun, T. (2026) https://BioRender.com/t0tb9cw).

Generally, mutations in tumour suppressor genes such as TP53 and CDKN2A/p16 reduce the cell’s ability to control growth or repair DNA damage, while mutations in oncogenes such as epidermal growth factor receptor (EGFR) and Kirsten rat sarcoma viral oncogene homologue (KRAS) drive uncontrolled cell division [6]. Over time, the combined effect of mutations in tumour suppressor genes and oncogenes transforms normal lung cells into cancer cells [7,8,9]. By the end of the promotion stage, cancer cells have acquired numerous mutations, providing them with a solid foundation for more advanced behaviours in the progression stage where further intracellular changes occur, which leads to metastatic tendency and allows possible resistance to therapy (Figure 1).

Apart from the increasing incidence and mortality rates of lung cancer, lung cancer drug resistance is a major factor that challenges the course of treatment in patients. Specific gene mutations, such as KRAS, have been shown to be correlated with acquired resistance in lung cancer cells [10]. Approximately 43–89% of NSCLC patients show overexpression of EGFR or mutations in intracellular EGFR, which can interfere with the standard treatment, as many drugs are ineffective against KRAS mutants [11]. Despite the targeted therapies successfully achieve initial treatment for EGFR-mutated NSCLC, drug resistance remains a near-universal barrier to long-term survival. Historically, first- and second-generation EGFR tyrosine kinase inhibitors (TKIs) yielded median progression-free survival (PFS) of only 9 to 13 months before disease progression presented—driven in approximately 50% to 60% of cases by the acquired EGFR T790M gatekeeper mutation [12]. Although the third-generation targeted drug for NSCLC, T790M-selective TKI osimertinib demonstrated superior PFS over platinum-pemetrexed chemotherapy in T790M-positive relapsed settings (10.1 vs. 4.4 months) and extended first-line PFS to 18.9 months compared to 10.2 months with standard TKIs, development of resistance ultimately persists [12,13]. Disease progression under osimertinib highlights an evolving spectrum of complex, heterogeneous resistance pathways, including tertiary EGFR mutations, off-target bypass, and persistent environmental pollution drivers, this multi-factorial resistance demands next-generation therapeutic strategies.

There are some significant factors that contribute to lung carcinogenesis, namely tobacco smoking and genetic predisposition. Interestingly, after tobacco smoking, rising ambient fine particulate matter (PM_2.5_) is one of the most significant contributing factors that has become even more prominent in recent years [14]. PM_2.5_ plays a role in increasing oxidative stress levels and inducing inflammatory responses, which collectively cause alterations within genetic compartments and eventually lead to lung carcinogenesis [15]. Crucially, PM_2.5_ pollution has emerged as a key player, driving risk of lung adenocarcinoma development in younger, non-smoking cohorts [16]. Driven by population aging and rising PM_2.5_ exposure, global lung cancer deaths are expected to reach 3 million by 2035, cementing the disease as a critical global public health challenge [1,2,16,17,18].

## 2. PM_2.5_: The Factor of Lung Carcinogenesis

PM_2.5_ refers to particles that have a diameter of 2.5 micrometres or smaller. These particles are small enough to enter the alveoli via inhalation and penetrate the bloodstream through the alveolar–capillary barrier, which allows ≤0.5 micrometre particles to pass through for gas exchange [19,20]. On the other hand, particles larger than 0.5 micrometres depend on different modes of endocytosis to enter the blood circulation [20]. Currently, there is a global problem regarding rising PM_2.5_ levels, as there are not many countries in which PM_2.5_ levels are in line with World Health Organization (WHO)’s recommendations. Globally, PM_2.5_ was around 32.8 μg/m^3^ during the period from 2000 to 2019, which exceeds the recommended level of no more than 5 μg/m^3^ provided by the WHO [21]. According to the data from the Global Burden of Disease Study (GBD), there is an increasing number of patients being diagnosed with lung cancer who have no prior smoking history and are at high risk of being exposed to ambient PM_2.5_ [22]. Moreover, PM_2.5_ is reported to be linked to an increase in lung cancer mortality, which has risen by 21.6% from 1990 to 2017, in addition to a 12.8% increase in disability-adjusted life years (DALYs) in the same period [23]. The studies in developing countries regarding PM_2.5_-induced lung cancer remain relatively scarce. A nationwide study in China investigated the positive association between PM_2.5_ exposure and the annual rates of lung cancer incidence in both males and females [24]. Safiri et al. (2021) demonstrated that ambient PM_2.5_ exposure contributes to 15.3% of the occurrence rates of lung cancer, which is only second to the contribution of cigarette smoking (62.4%) [25]. The analysis from Thaichana et al. (2026) found the positive association between average daily PM_2.5_ exposure and lung adenocarcinoma incidence in upper northern region of Thailand from 2013 to 2017 where seasonal biomass burning causes elevated PM_2.5_ levels, highlighting the long-term effects of PM_2.5_ on lung cancer incidence [16]. Moreover, the study cohorts in UK Biobank revealed that high exposure to PM_2.5_ is significantly associated with higher risk of lung cancer [26]. Similarly, the study from seven European cohorts showed that long-term exposure to PM_2.5_ is correlated with an increase in lung cancer incidence [27]. Furthermore, with the same cohorts, the longitudinal study demonstrated that the higher risk of lung cancer mortality is linked to prolonged exposure to PM_2.5_ [28]. However, these cohorts retain a limitation in that they did not entirely exclude those with smoking history which could present as a potential factor of predisposition. Taken together, PM_2.5_ is becoming a critical factor to consider in risk of lung cancer development.

The sources of PM_2.5_ in general include combustion processes, construction, and agricultural activities [29]. In combustion processes, PM_2.5_ is primarily produced from the exhaust smoke of automobiles or other vehicles, factory or coal power plants, and the burning of biomass. Construction sites that involve dust generation and agricultural practices that involve field burning also contribute to the production of PM_2.5_. Exposure to PM_2.5_ is considered a health hazard as it can irritate the respiratory tract, resulting in coughing, throat irritation, and shortness of breath, which can worsen respiratory conditions such as asthma or chronic obstructive pulmonary disease (COPD) [30,31]. PM_2.5_ also triggers local inflammation in lung tissue and weakens the natural defence mechanisms of the lung through mitochondrial dysfunction and ROS upregulation, making it more prone to respiratory infections such as bronchitis and pneumonia [32,33]. Long-term exposure to PM_2.5_ can induce systemic inflammation and increase oxidative stress as a result of the increase in ROS in the system. Moreover, PM_2.5_ can enter the bloodstream, where heavy metal components can induce oxidative stress through ROS production, increasing the risk of serious cardiovascular issues [34]. Furthermore, recent studies suggest that persistent exposure of PM_2.5_ has been linked to a higher risk of lung cancer, as chronic exposure to ROS can prolong inflammatory response, and sustained tissue damage plays a significant role in the development of lung cancer [35,36].

The main contributor to the pathogenesis of disease related to PM_2.5_ exposure is its ambient components, such as polycyclic aromatic hydrocarbons (PAHs), pollutants coming from natural or anthropogenic sources in the environment [37,38,39]. As microcontaminants, PAHs contribute to genotoxicity and subsequently cause mutagenesis [17,39]. In general, KRAS and EGFR are two significant genes that are mutated in patients with lung cancer [17,40]. The genetic profiles of nonsmokers who develop lung cancer have shown evidence of the mutation of EGFR and TP53, which have been identified as common drivers [15,41]. Chen et al. (2020) stated in a proteogenomic study of lung cancer in East Asian nonsmokers that there is a link between EGFR and TP53 mutations and exposure to PM_2.5_ and PAHs [42]. With such emphasis on the relationship between genetic alterations and environmental pollutants, it is most likely that the pathogenesis of lung cancer is strongly associated with exposure to pollution with concentrations of PM_2.5_.

## 3. NLRP3 Inflammasome and PM_2.5_ in Lung Cancer

**Figure 2:**
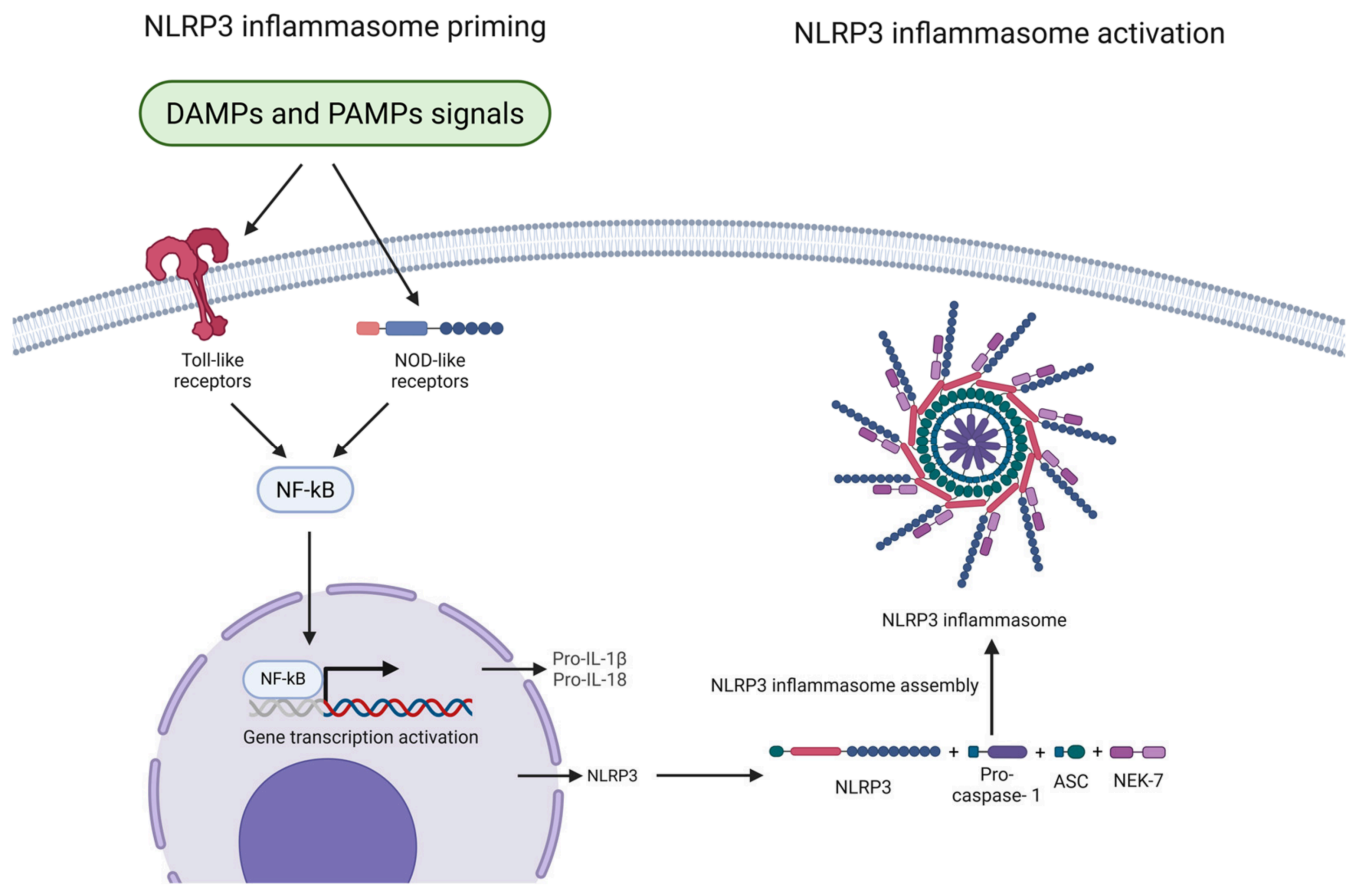
The schematics representing the priming and the activation of the NLRP3 inflammasome. The priming signal induced by the recognition of DAMPs or PAMPs, while the second signal allows the assembly of the inflammasome and its subsequent activation. (Created in BioRender. Worawichitchaikun, T. (2026) https://BioRender.com/t0tb9cw).

The NOD-, LRR- and pyrin domain-containing protein 3 (NLRP3) inflammasome is one of the most studied inflammasomes, characterised as a cytosolic NOD-like pattern recognition receptor protein complex that acts in response to cellular stress or cell membrane damage [43]. Subdivided into a NOD-like receptor family, the assembly of the inflammasome triggers the activation of caspase-1, leading to the generation of proinflammatory cytokines such as interleukin-1β (IL-1β) and interleukin-18 (IL-18) [44]. The expression of NLRP3 at basal levels is not sufficient to activate the inflammasome; therefore, two major processes are required for the activation of the NLRP3 inflammasome: priming and activation [45,46,47]. The first signal, priming, is induced by the recognition of damage-associated molecular patterns (DAMPs) or pathogen-associated molecular patterns (PAMPs), which primes the expression of pro-IL-1β and pro-IL-18, and the second signal is subsequently induced by the activated NLRs, which trigger the assembly of the inflammasomes, in turn activating caspase-1 [48,49,50] (Figure 2). The mature caspase-1 then mediates the activation of these inflammatory cytokines, subsequently generating the bioactive IL-1β and IL-18 and promoting processes of inflammation [51]. Therefore, the activation of caspase-1 causes cell death via pyroptosis, an inflammatory cell death linked to several cases of disease incidence [49,50,51,52,53].

A clinical study demonstrated that NLRP3 protein expression on CD3^+^ cells in early-stage NSCLC patients was higher than that of healthy individuals and could potentially be applied as a biomarker for discriminating the two conditions [54]. Similarly, plasma levels of NLRP3 in NSCLC patients were reported to be higher than those of the healthy control group [55]. The clinical evidence to support the relevance of NLRP3 in lung cancer is provided by large-scale epidemiological data and genomic profiling. Multi-cohort genomic investigations, including data from the Harvard Lung Cancer Susceptibility (HLCS) study and the PLCO Screening Trial (International Journal of Cancer), have mapped single nucleotide polymorphisms (SNPs) across core NLRP3 inflammasome pathway genes [56]. These studies revealed that specific germline genetic variants governing baseline NLRP3 signalling efficiency serve as strong, independent predictors of overall survival (OS) and recurrence-free survival (RFS) in NSCLC patients [56]. Complementing these systemic findings, a secondary analysis of the landmark CANTOS trial, which originally evaluated canakinumab in patients with atherosclerosis, demonstrated that targeting the primary downstream product of the NLRP3 inflammasome, IL-1β, significantly impacts lung cancer development [57]. Specifically, treatment with high-dose canakinumab, 300 mg every three months, led to a dose-dependent 67% reduction in lung cancer incidence and a 77% reduction in lung cancer mortality [57].

According to prior studies, the primary molecular mechanism of PM_2.5_-induced lung damage is the upregulation of ROS and mitochondrial dysfunction in both bronchial epithelial cells and macrophages [58,59]. Mechanistically, PM_2.5_ induces superoxide anion production at the electron transport chain, which leads to oxidative phosphorylation, mitochondrial dysfunction, and inhibition of ATP synthesis, resulting in necroptosis or pyroptosis [58,60,61,62,63]. An increase in mitochondrial ROS (mtROS) production from mitochondrial dysfunction can free thioredoxin-interacting protein (TXNIP) from thioredoxin (TRX) and allow TXNIP to bind to NLRP3 protein. Additionally, mitochondrial dysfunction releases mitochondrial DNA (mtDNA), which is then further oxidised and drives NLRP3 inflammasome assembly, further facilitating the activation of caspase-1 and the maturation of pro-inflammatory cytokines such as IL-18 and IL-1β [61,64,65,66,67]. Moreover, the uptake of PM_2.5_ into the cell induces phagolysosomal dysfunction, leading to the release of the digestive enzyme cathepsin B, which is directly involved in the activation of the NLRP3 inflammasome [68,69]. The findings implicate that chronic exposure to PM_2.5_ can cause and sustain chronic inflammation, primarily through upregulation of ROS production and phagolysosomal dysfunction. Furthermore, IL-1β and IL-18 have been known to promote epithelial–mesenchymal transition (EMT), which is implicated in various aspects of cancer progression, including tumour initiation, invasion, metastasis, and therapy resistance [70,71]. These findings suggest that ROS-induced NLRP3 activation from PM_2.5_ exposure is one of the potential causes of lung cancer development and progression.

As previously mentioned, KRAS and EGFR gene mutations are common in lung cancer patients. KRAS mutation is reported to cause the elevation of mitochondrial function and increase in mtDNA in order to meet the demands of rapid tumour growth [72,73]. According to prior studies, the elevation of mtDNA copy number accelerates the progression of lung adenocarcinoma [74]. Similarly, EGFR mutation has been reported to be associated with mtDNA elevation and mutation, contributing to lung cancer progression and metastasis [75,76,77]. Furthermore, EGFR mutation has been shown to disrupt the TXNIP-TRX axis, leading to an increase in free TXNIP and a downstream effect of increasing oxidative stress and NLRP3 inflammasome activation [78,79,80]. This information corroborates evidence from prior studies demonstrating that mtDNA promotes NLRP3 inflammasome activation, while a reduction in mtDNA suppresses NLRP3 inflammasome activation in lung injury and inflammation, though not well-reported specifically in lung cancer [81,82].

## 4. PM_2.5_-Induced NLRP3 Inflammasome Activation as a Potential Concept in Lung Cancer Drug Resistance

Cancer drug resistance is a complex multifactorial process that allows cancer to evade and become tolerant to the effects of conventional drug therapy and renders the therapy ineffective [83]. Factors that are prominent in cancer drug resistance include the therapy, cancer cells, and the host environment, which can give rise to either intrinsic or acquired resistance [84]. The observation of cancer cells at their molecular level reveals that they are always evolving and under stress, which allows them to have various levels of response to therapy [84]. The production of ROS is shown to be associated with the resistance to long-term treatment with EGFR TKIs in lung cancer patients [85]. In general, EGFR is a trans-membrane protein in the family of receptor tyrosine kinases (RTKs) that facilitates signalling pathways to control cellular proliferation [86,87]. Evidence shows that there is a high level of basal ROS present in TKI-resistant NSCLC cell lines, which is associated with higher NADPH oxidase 2 (NOX2) expression [88]. Previous studies have also stated that the oxidation of Cys797 of EGFR stimulates NOX2 and enhances the production of ROS [89,90,91]. Moreover, the mutation of EGFR Cys797 that increases EGFR activity can yield abnormal phosphorylation and disruption of the dimer structure of EGFR, leading to TKI resistance in NSCLC cell lines [90,92]. ROS production can also be promoted by cigarette smoke and further induce EGFR TKI resistance in NSCLC cell lines, although the underlying mechanisms are yet to be elucidated [93].

In addition to the effect of ROS on EGFR TKIs in lung cancer patients, the role of PM_2.5_ in resistance to EGFR TKIs has also been investigated in some studies. It has previously been acknowledged that PM_2.5_ plays a crucial role in the pathogenesis of lung carcinoma, specifically with regard to EGFR overexpression and mutation [15,94,95]. A study by Chen et al. (2024) found that lung cancer patients who were nonsmokers, most of whom were women with EGFR mutations, exhibited increased expression of proteins associated with PAH or nitro-PAH exposure [15]. This suggests that PM_2.5_ may be correlated with various processes of cancer development, ranging from initiation to progression. Along with EGFR overexpression, PM_2.5_ exposure has a tendency to induce aryl hydrocarbon receptor (AhR)-dependent transcription of transmembrane serine protease 2 (TMPRSS2) and interleukin-18 (IL-18), which subsequently leads to the promotion of lung cancer progression in patients; this is correlated with the established AhR–TMPRSS2–IL-18 pathway [96]. From the particular experimental evidence, we see that the overexpression of EGFR in lung carcinogenesis and the activation of AhR in lung cancer progression, both by PM_2.5_, could offer an aspect in which PM_2.5_ might potentially induce lung cancer progression directly via these pathways. However, AhR activation could influence cancer progression independently through direct transcriptional upregulation of CYP1A1, facilitating PTEN inhibition, β-Catenin/Akt activation, and modulation of T-cell activation and polarisation [97,98,99,100].

**Figure 3:**
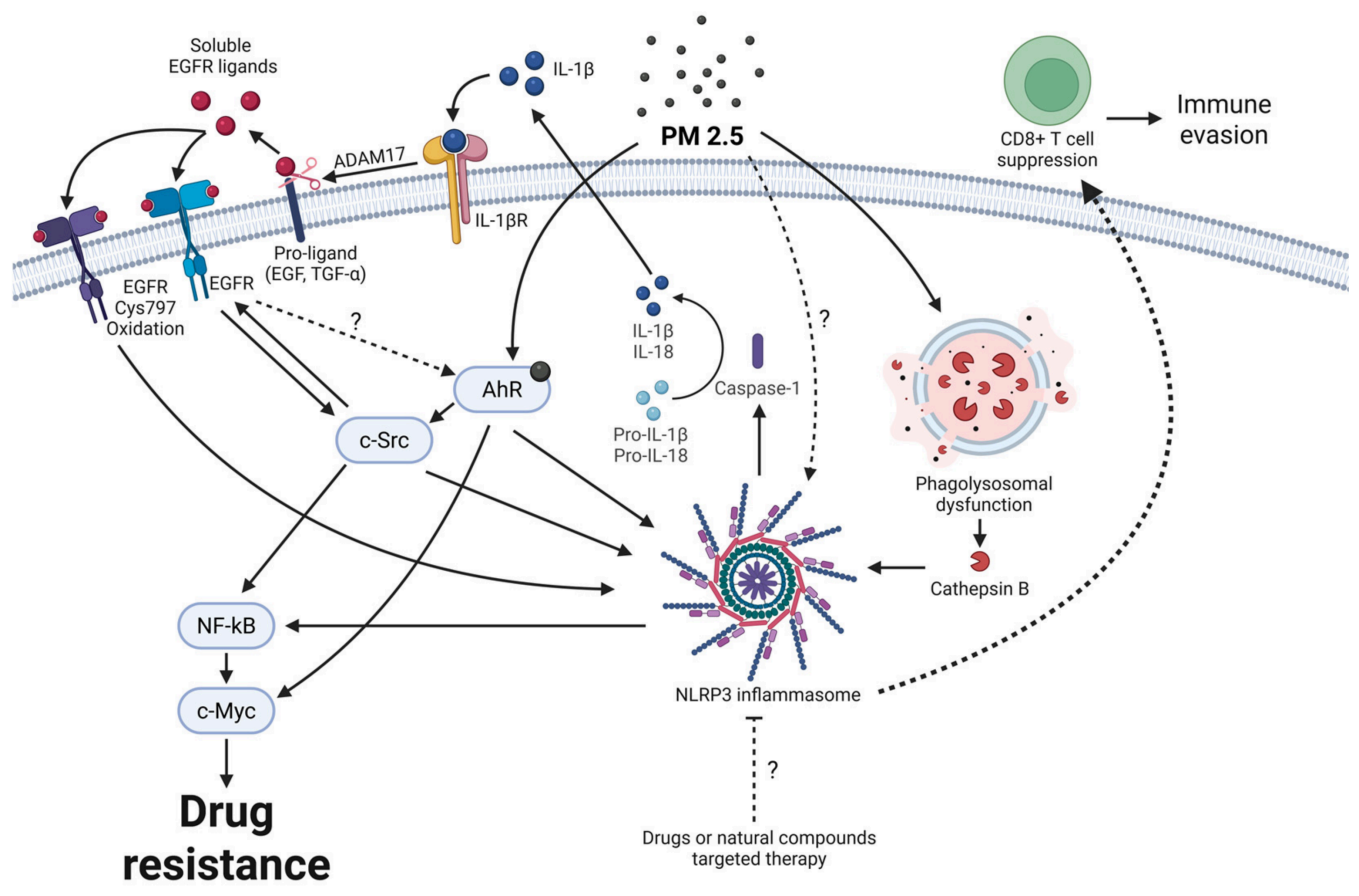
PM_2.5_ and NLRP3 in lung cancer drug resistance. PM_2.5_ exposure induces ROS production, AhR activation, and phagolysosomal dysfunction, which influence NLRP3 activation. NLRP3 activation enables the maturation of its correlating cytokines, especially IL-1β and IL-18, where IL-1β induces EGFR and subsequently gives rise to the crosstalk between EGFR and c-Src signalling. Cys797 oxidation provides a cascade for ROS upregulation where AhR activation leads to c-Src signalling, all of which contribute to cancer drug resistance. NLRP3 activation may lead to the suppression of CD8^+^ T cells, contributing to immune evasion and resistance to therapy. (Created in BioRender. Worawichitchaikun, T. (2026) https://BioRender.com/t0tb9cw).

Besides the activity of PM_2.5_-induced ROS, which can cause EGFR overexpression in patients with lung cancer, there have been suggestions regarding the ability of PM_2.5_ to induce lung cancer drug resistance to EGFR TKIs via the activation of AhR, which in turn activates proto-oncogene tyrosine-protein kinase Src (Src) signalling [101]. Exposure to PM_2.5_ and its ambient component, including PAHs, not only induces ROS and inflammatory processes but also gives rise to the crosstalk between EGFR and Src, which contributes to the resistance to EGFR TKIs and possibly other EGFR-targeted therapies [15,101]. Moreover, AhR also possesses the ability to bind with the promoter regions of cMyc, a gene that is known to participate in drug resistance [102]. Also, Vogeley et al. (2022) reported that EGFR has a critical role in AhR ligand-specific responses, which was identified via RNA-seq [103]. From the correlation between established pathways presented above, they implicate that there is a strong relationship between EGFR and AhR, along with ROS activity, helping to promote EGFR TKI resistance in patients with lung cancer, although the underlying mechanism of the hypothesised EGFR/AhR crosstalk from the perspective of cancer drug resistance is yet to be investigated and requires further experimental studies to determine whether the positive feedback of the crosstalk could enhance drug resistance in lung cancer (Figure 3).

As previously mentioned, NLRP3 plays a major role in initiating caspase-1 activation and driving IL-1β and IL-18 maturation, contributing to pyroptosis, an inflammatory form of programmed cell death [64,104,105,106,107]. Emerging evidence suggests that the major inflammatory cytokines activated by NLRP3, especially IL-1β, possess the ability to upregulate EGFR via Src and CXCL1–CXCR2-dependent pathways [108,109]. From this particular well-illustrated relationship between the pathways, this correlates with the suggested crosstalk between EGFR and Src, which may contribute to the resistance to EGFR TKI treatment. Another in vivo study demonstrated that mice exposed to PM_2.5_ show an increase in NLRP3 and EGFR expression, as well as increased levels of mRNAs IL-18 and CXCL1 [110]. From this proven in vivo study, it can be hypothesised that PM_2.5_-induced NLRP3 activation might give rise to EGFR TKI resistance via the crosstalk between EGFR and Src; however, similarly to EGFR–AhR crosstalk, the underlying mechanism remains unknown. Moreover, the NLRP3 inflammasome activity has been reported to promote resistance to immune checkpoint inhibitors (ICIs). Chen et al. (2018) stated that the upregulation of CD38 promotes resistance to anti–programmed cell death 1 (anti–PD-1) checkpoint blockade, reducing CD8^+^ T cell expression [111]. A study by Theivanthiran et al. also reported that the tumour-intrinsic NLRP3 inflammasome allows the recruitment of polymorphonuclear myeloid-derived suppressor cells (PMN-MDSCs) into the tumour microenvironment (TME) via the release of heat shock protein 70 (HSP70) which binds to toll-like receptor 4 (TLR4), causing the inhibition of CD8^+^ T cells and contributing to tumour immune evasion and resistance to immunotherapy [112]. The tumour-intrinsic NLRP3 inflammasome activation pathway has been further verified in other cell lines, including lung cancer models [112]. Based on inferred evidence from these previous studies, we hypothesise that the activation of NLRP3 inflammasome via PM_2.5_ may be responsible for the acquired immunoresistance in lung cancer. Further experimental investigations into these hypothesised mechanistic pathways are required to validate the theory and to explore strategies to suppress or utilise the inflammasome in cancer treatment. Taken together, the NLRP3 inflammasome could potentially be a target molecule for novel therapeutic strategies to overcome drug resistance and enhance chemotherapy response in lung cancer.

## 5. NLRP3 Inflammasome as a Double-Edged Sword

**Figure 4:**
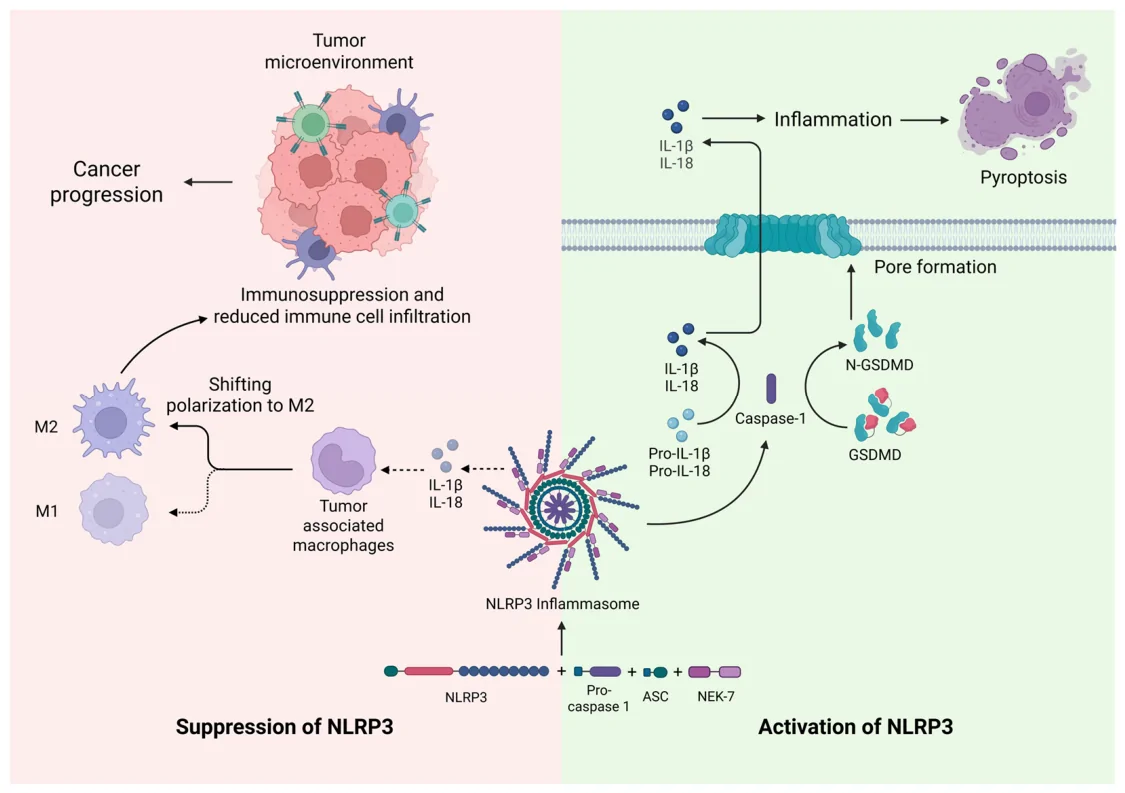
Alternating pathways depicting contradictory roles of NLRP3 inflammasome suppression and activation. Suppression of NLRP3 causes tumour-associated macrophages to favour M2 polarisation, leading to immunosuppression in the TME, which promotes cancer progression. Activation of NLRP3 allows mature caspase-1 to cleave GSDMD, leading to transmembrane pore formation, which induces pyroptosis. (Created in BioRender. Worawichitchaikun, T. (2026) https://BioRender.com/t0tb9cw).

According to prior studies, NLRP3 inflammasome activation contributes to molecular pathways associated with different stages of cancer progression, including cancer drug resistance. Nevertheless, we find that NLRP3 inflammasome activation also displays an opposite role in mediating cancer survival in certain conditions. For instance, the activation of caspase-1 triggered by the NLRP3 inflammasome has the ability to cleave gasdermin D (GSDMD), a principal member in the gasdermin protein family with a role in inducing pyroptotic cell death [113,114]. GSDMD initiates transmembrane pore formation and the release of inflammatory cytokines to damage cell membrane integrity, eventually resulting in cell death [115,116,117] (Figure 4). One study reported that saikosaponin-D, a bioactive compound found in Bupleurum chinense, induces the activation of the NLRP3/caspase-1/GSDMD pathway and the accumulation of ROS, leading to pyroptosis in NSCLC cells [118]. A study by Kang et al. (2024) stated that the NLRP3/caspase-1/GSDMD pathway was activated by PM_2.5_, resulting in pyroptotic cell death [119]. Another recent study revealed the mechanism of an NLRP3 agonist that binds to NLRP3 directly to induce pyroptosis and restore sensitivity to targeted therapy in cancer cells with drug resistance [120]. These research articles indicate that the expression of NLRP3 may trigger inflammatory cytokine production and drive pyroptosis, enhancing the efficacy of chemotherapeutic agents [104,118,121].

Moreover, total NLRP3 suppression represents a double-edged sword, as it can facilitate a macrophage polarisation shift from a pro-inflammatory M1 state to an anti-inflammatory M2 state. Pro-inflammatory cytokines, specifically IL-1β and IL-18, are crucial for maintaining an active M1 macrophage phenotype, which plays a major role in anti-tumour activity and host immune activation [122]. Shifting toward an anti-inflammatory M2 phenotype can lead to immunosuppression and a reduction in immune infiltration, particularly of CD8^+^ T cells, into the TME [123]. In a prior study, Lu et al. (2020) showed that M2 polarisation in tumour-associated macrophages (TAMs) led to cancer growth and metastasis in the co-culture of THP-1 monocytes and the A549 lung cancer cell line, and in syngeneic murine tumour models [124] (Figure 4). Cheng et al. (2022) stated that suppression of NLRP3 inflammasome activity in TAMs can induce cisplatin resistance in multiple cancer types, including lung cancer, breast cancer and head and neck cancer [125]. Nevertheless, clinical evidence regarding the prognostic value of specific TAM densities in NSCLC remains contradictory, as intratumoral M1 density is positively correlated with extended patient survival, while M2 densities show no significant association with survival time [126]. Moreover, Gu et al. (2022) reported the opposite pattern in pancreatic ductal adenocarcinoma, where NLRP3 activation in TAMs drove M2-like polarisation and promoted lung metastasis [127]. Consistent with Amo-Aparicio et al. (2023), NLRP3 activation reduced cytotoxic CD8^+^ T-cell activation and increased tumour expansion, while the combination of gemcitabine with the orally active NLRP3 inhibitor OLT1177 increased the efficacy of monotherapy [128]. Similarly, Chen et al. (2023) demonstrated that NLRP3 expression within the TAMs of HNSCC correlated with M2 polarisation and markedly worse overall survival [129]. Considering these two contrasting views, we believe that a certain threshold of inflammation appears to be necessary to either induce drug resistance in lung cancer or trigger cancer cell death effectively. In addition, cancer types and their respective mechanistic interactions with NLRP3 may be responsible for the different outcomes yielded from the expression of the NLRP3 inflammasome. Taken together, this contrasting evidence suggests that NLRP3 could be a potential therapeutic target, depending on the context of specific tumour stage, tumour type, and the baseline immune architecture to avoid accelerating immune evasion or chemoresistance.

## 6. Targeting NLRP3 Inflammasome and Inflammatory Cytokines as a Therapeutic Regimen for Lung Cancer

Numerous therapeutic regimens have been used in lung cancer patients with cancer drug resistance. When considering the NLRP3 inflammasome and its related cytokines as potential targets, there have been developments in such therapies that target the inflammasome itself or its correlated inflammatory cytokines. For instance, a diarylsulfonylurea MCC950 selectively binds to NLRP3 to prevent its oligomerisation and the activation of caspase-1, consequently reducing the maturation and secretion of IL-1β and IL-18 [130,131]. Another example is OLT1177, a β-sulfonyl nitrile that inhibits the activity of NLRP3 ATPase and similarly reduces IL-1β and IL-18 levels [132]. Recently, OLT1177 was evaluated as an oral NLRP3 inhibitor in a phase 2A trial for acute gout patients, with a positive result [133,134]. MCC950 was also shown to have a cardioprotective effect and improve cardiac function in separate studies on mouse models [135,136]. In a type-2 diabetic rat model, MCC950 was able to alleviate retinopathy via the ROS-NEK7-NLRP3 pathway [137]. However, the issue regarding potential hepatotoxicity and off-target tendency of MCC950 led to a halt in clinical trials [138,139]. Despite clinical trials investigating other inflammatory diseases, there is currently no evidence on utilising NLRP3 inhibitor as a therapeutic regimen for cancer. In addition to NLRP3 inflammasome blockade, pre-clinical and early-phase trials have begun investigating different NLRP3-related molecules. Randomised trials utilising canakinumab, whose role is the inhibition of IL-1β in atherosclerosis, have been performed and achieved positive results in reducing lung cancer mortality [57]. Simultaneously, a phase-II study from Zhang et al. (2023) found that canakinumab treatment showed favourable efficacy in lung cancer patients with high-risk lung nodules [140].

In addition to various trials in experimental stages, certain natural products and their related extracts are being examined for their use in treating cancer in various stages. As mentioned in previous sections, lung cancer drug resistance present similar correlating pathways of PM2.5/NLRP/ROS-induced processes to those of cancer progression. Therefore, there is a strong possibility that utilising treatment regimens to manipulate specific pathways may help alleviate and potentially inhibit cancer drug resistance. Natural products have been shown to exhibit anti-inflammatory properties that are correlated with the nuclear factor erythroid 2-related factor 2 (Nrf2) pathway and its downstream antioxidant targets [141]. Natural compounds, including sulforaphane, xanthohumol, and quercetin, have been reported to induce the Nrf2 pathway and inhibit NLRP3 inflammasome activation and formation in diabetic retinopathy, LPS-induced acute lung injury, alcohol-induced liver injury, and ulcerative colitis models [142,143,144,145]. Considering the roles of NLRP3 inhibitor in multiple clinical studies, its effects are mechanistically plausible in lung cancer treatment, which are not well-documented in oncology and are worth further investigation. In addition, natural compounds could display potential when combined with existing chemotherapeutic drugs to offer a promising approach towards the treatment of lung cancer.

## 7. Discussion

In recent years, PM_2.5_ has become a major concern regarding the process of lung carcinogenesis and its progression, including drug resistance, via the activation and accumulation of ROS. It has been established that persistent exposure to PM_2.5_ is strongly correlated with a higher risk of lung carcinogenesis, as prolonged inflammatory response and tissue damage play a significant role in the development of lung cancer. While PM_2.5_ can cause chronic inflammation systematically, the lungs are the most affected due to their role as a primary site of pollutant inhalation. The correlation between PM_2.5_ exposure and the activation of the NLRP3 inflammasome in further enhancing the process of lung carcinogenesis has been a subject of study, considering that the NLRP3 inflammasome is the main mediator of IL-1β and IL-18, both of which are linked to several cases of chronic inflammatory disease incidence. A study in which NLRP3 inflammasome is responsible for the recruitment of immunosuppressive cells in the TME has shown to facilitate tumour immune evasion and resistance to immunotherapy [115]. However, the direct association between PM_2.5_–ROS–NLRP3 axis and lung cancer drug resistance has yet to be elucidated and remains a knowledge gap. Further investigation of this conceptual molecular pathway could be a valuable insight in establishing PM_2.5_ as a key driver of NLRP3 activation which could lead to lung cancer drug resistance. For instance, an in vivo experiment conducting by exposing PM_2.5_ to test subjects and measuring the threshold of NLRP3 expression, correlated with lung cancer progression and drug resistance markers. Additionally, retrospective studies in lung cancer patients, specifically those who are nonsmokers and with high risks of exposure to PM_2.5_, could strengthen the causality of PM_2.5_ in lung carcinogenesis. Patients who develop drug resistance could further be tested for NLRP3 expression to determine its involvement in the process.

As previously proposed, PM_2.5_-induced NLRP3 activation could be critical in promoting the ability of the cancer to resist EGFR TKI treatment. Moreover, studies into the aspect of immunoresistance in lung cancer could enhance further understanding as NLRP3 activation within the lung cancer cells leads to CD38 upregulation which establish an immunosuppressive environment that facilitates immune evasion, similarly to the suppression of CD8^+^ T cells in various cancer model, thereby driving lung cancer resistance and potentially lung cancer recurrence [115]. The roles of pro-inflammatory cytokines such as IL-1β and IL-18 from NLRP3 inflammasome activation in inducing the EMT and intensifying local immunosuppression to accelerate disease relapse could also be studied as possible downstream mechanisms to prevent further exacerbation of the disease. Interestingly, there are contrasting views on the role of NLRP3 inflammasome activation which discusses whether it induces lung cancer resistance or facilitates pyroptosis through GSDMD-mediated pore formation at the cell membrane and enhances the activity of chemotherapeutic agents [118,119,120]. Furthermore, clinical evidence suggests that stages and types of cancer could influence NLRP3 mechanisms in inducing M2 polarisation and the progression of cancer. As previously mentioned, a certain threshold of inflammation correlated with NLRP3 activation and its cytokines suggests that the inflammasome is a double-edged sword in mediating drug resistance in cancer, although more evidence is needed to verify this. We believe that high-intensity, acute cellular stress triggers massive assembly of the NLRP3 complex, driving hyperactivation of caspase-1 and extensive cleavage of GSDMD. Cleaved GSDMD forms lytic plasma membrane pores, inducing cell swelling, rupture, and immunogenic pyroptotic death that can stimulate anti-tumour T-cell responses. In contrast, prolonged exposure to ambient PM_2.5_ induces low-level, sub-lytic NLRP3 signalling. Under this regime, caspase-1 cleavage remains below the threshold required for pore-mediated membrane lysis, permitting continuous secretion of mature IL-1β and IL-18 without inducing cell death. Furthermore, malignant epithelial cells frequently undergo epigenetic silencing or down-regulation of GSDMD, effectively uncoupling cytokine processing from cell death. This allows cancer cells to escape pyroptosis while exploiting paracrine IL-1β signalling to drive STAT3 activation, stemness, and acquired TKI resistance within the local microenvironment. From this, we hypothesise that NLRP3 might play an important role in lung cancer drug resistance and could serve as a potential target for developing novel therapeutic regimens that could overcome this challenge.

In light of advancements in therapeutic regimens for cancer drug resistance, there are still unresolved issues that require further understanding of the cellular, molecular, and environmental correlation between cancer and the mechanisms by which they adapt to conventional therapy. Prior clinical studies have shown a significant increase in NLRP3 protein expression in patients with early-stage NSCLC when compared with healthy individuals [54,55]. NLRP3-targeted therapy could reduce the likelihood of patients developing drug resistance when administered intermittently or in combination with a conventional therapeutic regimen. This remains a major gap in experimental and clinical research that requires extensive examination and validation. Aside from the considerations of dosage and treatment duration of NLRP3-targeted therapy, we also believe that the time point of alternating modalities should be taken into consideration to ensure minimal adverse events. However, the primary limitation is that systemic inhibition carries distinct clinical risks. Because baseline NLRP3 activity is crucial for innate immune defence against bacterial and fungal pathogens, continuous systemic blockade heightens the risk of opportunistic infections. Moreover, in immunocompetent tumour microenvironments, complete NLRP3 inhibition risks blunting immunogenic cell death (ICD) and reducing the efficacy of concurrent ICIs that rely on inflammasome-driven antigen presentation. Also, there are only a few variations of NLRP3 inhibitor that are under clinical and pre-clinical investigation, one of which was removed from clinical trials due to concerns regarding hepatotoxicity and off-target tendency. Research needs to address concerns regarding unforeseen adverse effects such as drug–drug interactions and toxicity burden for the patients when an NLRP3-targeted drug is used in combination with conventional treatment. To maximise therapeutic index while minimising adverse effects, precision treatment strategies must incorporate patient selection. For instance, in the aspects of environmental and biomarker stratification, systemic or inhaled NLRP3 inhibitors should be restricted to NSCLC patients with documented chronic exposure to high ambient PM_2.5_ levels and elevated baseline inflammatory markers. In the aspects of genomic and subtype selection, candidates should be enriched for oncogene-addicted subsets, such as those with EGFR-mutant NSCLC undergoing TKI therapy, where sub-lytic inflammatory signalling actively fuels resistance, rather than unselected cohorts receiving primary immunotherapy. In addition, localised delivery should be taken into consideration in that utilising aerosolised or inhaled formulations of NLRP3 inhibitors should achieve therapeutic concentrations within bronchial tissues while avoiding systemic immunosuppression. Further investigation into the NLRP3 inflammasome could validate its potential as a target molecule for novel therapeutic strategies to overcome the problems of drug resistance in lung cancer.

## 8. Conclusions

Lung cancer is one of the most prominent types of cancer, with high incidence and mortality rates worldwide. There is an increasing incidence rate of the disease in patients with younger age and a persistent resistance to therapy. The relationship between exposure to PM_2.5_ and its ambient component and the increased production of ROS has been illustrated, highlighting the significance of PM_2.5_-induced ROS, which plays multiple roles in several inflammatory diseases, including cancer. Reports show a rising incidence of lung cancer cases among nonsmokers particularly those exposed to high levels of PM_2.5_. NLRP3 activation from sustained exposure to PM_2.5_ is one of the important contributors to the development and progression of lung cancer. Moreover, PM_2.5_ exposure has been linked to the development of drug resistance in lung cancer, specifically to EGFR TKI treatment. PM_2.5_-induced NLRP3 activation could be critical in promoting the ability of the cancer to resist EGFR TKI treatment via the relationship between EGFR, AhR, Src, and ROS expression or establish an immunosuppressive environment that facilitates immune evasion. We find that a threshold of inflammation correlated with NLRP3 activation mediates the resulting outcomes in the progression of cancer, and a dedicated study into the roles of NLRP3 may help establish a novel approach in cancer therapy. Therefore, the overlapping correlation between PM_2.5_, ROS, NLRP3, and lung cancer progression and drug resistance could be essential in further studies. With experimental investigation and clinical validation, NLRP3-targeted therapy could potentially be beneficial to overcome the issue of lung cancer progression and drug resistance.

## Data Availability

No new data were created or analysed in this study. Data sharing is not applicable to this article.
